# Structure-Based Comparative Analysis Reveals the Landscape of Powdery Mildew Secretomes Across Five Genera

**DOI:** 10.3390/pathogens15060612

**Published:** 2026-06-08

**Authors:** Noman Ali, Nan Wu, Engin U. Akkaya, Mahinur S. Akkaya

**Affiliations:** 1 School of Bioengineering, Dalian University of Technology, Dalian 116024, China; nomanali@mail.dlut.edu.cn (N.A.); wu_nan@mail.dlut.edu.cn (N.W.); 2 State Key Laboratory of Fine Chemicals, Department of Pharmaceutical Engineering, Dalian University of Technology, Dalian 116024, China; eua@dlut.edu.cn

**Keywords:** powdery mildew, secretome, structural landscape, AlphaFold2, RNase-like proteins associated with haustoria (RALPH)

## Abstract

Powdery mildew fungi are major obligate biotrophic plant pathogens, that cause widespread disease in agricultural and natural ecosystems worldwide, but a comparative structural view of their secretomes across multiple genera has remained limited. Here, we performed computational structure prediction and comparative analysis of 7545 secretome candidates from 26 isolates representing five genera (*Blumeria*, *Erysiphe*, *Golovinomyces*, *Parauncinula*, and *Podosphaera*) using AlphaFold2-based structure prediction, structural annotation against CATH, SCOPe, and PDB, Foldseek-based clustering and network analysis, structure-based grouping of RALPH (RNase-like proteins associated with haustoria) candidates, and comparison with defined fungal effector structural families. The predicted secretomes showed comparable model confidence across isolates and revealed a conserved structural core composed of recurrent microbial ribonuclease, immunoglobulin/fibronectin-like, glycosidase-related, and other enzyme-associated folds, with MoHrip2-like representing the most prominent shared fold among defined fungal effector structural families. Structural clustering and network analysis identified a prominent RALPH-centered component with additional conserved and lineage-enriched communities. RALPH candidates formed a structurally diverse repertoire that could be partitioned into 15 topology-defined groups, several linked to previously characterized powdery mildew effectors. *Blumeria* was structurally distinct, showing expansion of RALPH-associated components and the absence of multiple fold/domain categories retained in dicot-associated genera. Together, these results establish a comparative structural landscape of powdery mildew secretomes and provide a framework for future functional, evolutionary, and genomics-driven studies of conserved and lineage-associated secretome candidate.

## 1. Introduction

Powdery mildew fungi are obligate biotrophic plant pathogens that establish intimate associations with living host tissues and cause economically important diseases on a wide range of crops and other plants [1,2]. A hallmark of this lifestyle is the formation of haustoria, specialized infection structures that mediate nutrient uptake and serve as major sites for deployment of the fungal secretome, including secreted effectors, at the host–pathogen interface [3,4]. Fungal secretomes therefore represent a key molecular layer underlying host colonization, immune suppression, and pathogenic specialization [5]. Understanding the composition and diversification of powdery mildew secretomes is thus essential for clarifying how these fungi maintain biotrophy and adapt to different host lineages. This importance is underscored by comparative genomic analyses showing that powdery mildew evolution is associated with genome expansion, extensive retrotransposon proliferation, and gene loss linked to obligate biotrophy [6]. Subsequent genome analyses in wheat powdery mildew further revealed a large effector complement and rapid diversification within *Blumeria* lineages [7].

A growing number of powdery mildew candidate effectors have now been identified, although only a subset has been experimentally characterized and functionally validated, particularly in wheat and barley powdery mildew systems, where functional studies have linked individual effectors to avirulence or virulence-associated activities [1,8]. Among these, RALPH (RNase-like proteins associated with haustoria) has emerged as one of the most prominent structural classes, particularly in *Blumeria* and related cereal powdery mildew systems [9]. Early large-scale analyses of *Blumeria* candidate secreted effector proteins also pointed to haustorial enrichment, family expansion, and widespread ribonuclease-like structural affinities within cereal powdery mildew effector repertoires [10]. Structural studies have shown that AVR_A6_, AVR_A7_, AVR_A10_, AVR_A13_, AVR_A22_, AVRPM2, and BEC1054 adopt related RNase-like scaffolds despite substantial sequence divergence [11,12,13,14], highlighting a level of structural conservation that is not readily apparent from sequence alone. Several of these proteins function as avirulence effectors recognized by barley immune receptors, including MLA6, MLA7, MLA10, MLA13, and MLA22, or by the wheat immune receptor PM2 [11,13,14,15], whereas BEC1054 has been characterized as a ribonuclease-like virulence effector that represses plant immunity [12,16]. Together, these findings establish RALPHs as a central framework for understanding powdery mildew effector biology. However, current structural knowledge is still derived largely from *Blumeria* and closely related cereal powdery mildew systems, leaving unresolved how broadly RALPH-related structures extend across other powdery mildew genera and whether additional recurrent structural classes also make major contributions to powdery mildew secretomes. This taxonomic bias is consistent with the deeper genomic characterization available for cereal mildew systems, including chromosome-scale evidence for a highly dynamic effector repertoire in wheat powdery mildew [17].

Recent advances in structural biology and protein structure prediction have expanded fungal effector research beyond sequence-based analysis alone. Structure-guided analyses have shown that many effectors with little or no detectable sequence similarity can still be grouped into recurrent structural families, providing a comparative framework for understanding effector evolution and diversification [18,19]. In *Magnaporthe oryzae*, computational structural genomics uncovered both common folds and previously unrecognized effector families, with subsequent work on the MAX superfamily demonstrating that a conserved β-sandwich core accommodates extensive diversification in disulfide patterns, terminal extensions, and surface properties [18,20]. The same pathosystem also yielded the ZiF (zinc-finger fold) family of secreted effectors, which is distributed across blast fungus lineages infecting multiple host species [21]. Similarly, combined experimental and computational analyses in *Fusarium oxysporum* f. sp. *lycopersici* defined the FOLD (*Fusarium oxysporum* dual-domain) effector class and further indicated that this pathogen deploys effectors built from a limited number of recurrent structural folds [22]. More broadly, recent structure-guided syntheses have consolidated multiple recurrent families, including LARS (*Leptosphaeria* Avirulence and Suppressing), MAX, FOLD, KP4/KP6-like, ToxA-like, Tin2-like, ZiF, and MoHrip2-like, and have emphasized that structural comparison can reveal both widely recurring folds and lineage-restricted architectures that are often not apparent from sequence data alone [19]. At the same time, functional effector studies in non-*Blumeria* powdery mildew systems are increasing. Examples include *Erysiphe necator* effectors CSEP080 and CSEP087, effector candidates identified from the haustorial transcriptome of *Erysiphe pisi*, and the *Podosphaera xanthii* effectors PxLPMO1 and PxCDA3, which together indicate that effector repertoires outside cereal mildew systems are biologically diverse but remain much less structurally characterized [23,24,25,26,27]. Against this background, a broad structural view of powdery mildew secretomes is particularly needed to determine whether their effector repertoires are dominated primarily by the RALPH framework or whether additional recurrent structural classes also make substantial contributions across genera.

Our previous sequence-based comparative analysis of powdery mildew secretomes across 26 isolates from five genera identified both conserved core secretome candidates and host lineage-associated divergence [28]. However, sequence-based comparisons alone are unlikely to fully capture effector similarity in powdery mildew fungi, particularly for rapidly evolving yet structurally conserved classes such as RALPHs. More broadly, recent studies have shown that fungal effectors with little apparent sequence similarity can still share common folds and be organized into recurrent structural families [19,29]. In the present study, we used AlphaFold2-based structure prediction to analyze 7545 powdery mildew secretome candidates from 26 isolates representing five genera: *Blumeria*, *Erysiphe*, *Golovinomyces*, *Parauncinula*, and *Podosphaera*. We combined structural annotation, clustering, network analysis, focused grouping of RALPH-related candidates, and comparison with defined fungal effector structural families to establish a comparative structural landscape of powdery mildew secretomes. This framework was used to identify conserved structural features shared across genera, characterize the structural diversification of the RALPH repertoire, and examine lineage-associated differences among powdery mildew secretomes, while noting that host association and genus are partially coupled in the present sampling.

## 2. Materials and Methods

### 2.1. Secretome Candidate Dataset and AlphaFold2 Structure Prediction

This study was based on the standardized powdery mildew secretome dataset established in our previous comparative analysis [28], which included 26 isolates representing five genera: *Blumeria*, *Erysiphe*, *Golovinomyces*, *Parauncinula*, and *Podosphaera*. The 7545 powdery mildew secretome candidates analyzed in this study are listed in Table S1.

Protein structures of the 7545 secretome candidates were predicted using AlphaFold2 implemented through the LocalColabFold platform [30,31]. Mature protein sequences, defined as sequences after removal of the predicted signal peptide, were used as input. For each query protein, five models were generated, and the model with the highest predicted local distance difference test (pLDDT) score was retained for downstream analyses. Model confidence was evaluated using pLDDT and predicted template modeling (pTM) scores. Protein structures were visualized and edited in PyMOL 2.3.4 (https://www.pymol.org/, accessed on 3 March 2026).

### 2.2. Structural Annotation Against CATH, SCOPe, and PDB

To annotate the predicted structural space of the powdery mildew secretome, the 7545 AlphaFold2 models were searched against the CATH50, SCOPe40, and PDB databases using locally installed Foldseek (release 8-ef4e960) [32,33,34,35]. Hits were retained if they satisfied either of the following criteria: (i) alignment TM-score (alntmscore) ≥ 0.5 and E-value ≤ 1 × 10^−5^; or (ii) alntmscore ≥ 0.5, 1 × 10^−5^ < E-value < 1, and target-normalized TM-score (ttmscore) ≥ 0.5. Secretome candidates with at least one retained hit in a given database were considered structurally annotated in that database, whereas those without retained hits were considered unannotated. Multiple retained hits from the same query were allowed, and all annotations meeting the above criteria were retained and recorded in Table S1. For CATH and SCOPe, retained hits were summarized as fold/domain annotation distributions across isolates. Accordingly, the CATH and SCOPe summary plots report retained annotation-hit counts rather than one-best-hit assignments per protein. For PDB, retained hits were used as reference structural annotations to aid interpretation of secretome candidates and structural clusters.

### 2.3. Structure Cluster Analyses

To define the global structural organization of the powdery mildew secretome, the 7545 predicted structures were clustered using the easy-cluster module of Foldseek (release 10-941cd33) [35], with an E-value cutoff of < 1 × 10^−5^, TM-score normalized by alignment length, and alignment TM-score ≥ 0.5. All other parameters were kept at default settings. Structural clusters were ranked by the number of members in each cluster. Representative structures were the cluster representatives directly selected by Foldseek during easy-cluster analysis.

A cluster abundance matrix was then constructed, in which rows represented structural clusters, columns represented isolates, and each cell contained the number of secretome candidates from a given isolate assigned to the corresponding cluster. Principal component analysis (PCA) was performed using this raw cluster abundance matrix to summarize variation in structural cluster repertoires among 26 isolates. PCA was performed using the OECloud tools (https://cloud.oebiotech.com, accessed on 3 March 2026).

To further examine structural relationships among abundant clusters, we constructed a network from representative proteins sampled from the 92 largest structural clusters. For each cluster, the 20 proteins with the highest pLDDT scores were selected, resulting in a total of 1840 proteins. All-versus-all pairwise structural similarities among these proteins were calculated using Foldseek, and edges with alntmscore < 0.5 were removed. The resulting network was imported into Gephi 0.10.1 [36], with alntmscore used as the edge weight, and visualized using the Fruchterman–Reingold layout algorithm until a stable configuration was obtained.

### 2.4. Structural Comparison with Reference Effector Structures

The 7545 predicted secretome structures were compared against two reference structure sets: known powdery mildew effectors (Table S2) and defined fungal effector structural families (Table S3). The known powdery mildew effector set comprised 75 AlphaFold2-predicted effector structures and additionally included experimentally determined PDB structures when available. The defined fungal effector structural family set was compiled with reference to previous structure-based studies of fungal effectors [29,37] and comprised 40 effectors represented by 56 PDB chains from 13 structural families, namely AvrSr35-like, AvrSr50-like, FOLD, KP4-like, KP6-like, LARS, MAX, MoHrip2-like, NTF-like, PevD1-like, Tin2-like, ToxA-like, and ZiF. Structural similarity searches were performed using Foldseek (release 8-ef4e960) [35] and re-evaluated using US-align [38]. For both reference sets, only matches supported by Foldseek alntmscore ≥ 0.5 and US-align TM-score ≥ 0.5 were retained. Retained matches were recorded in Table S1.

### 2.5. Structure-Based Grouping of RALPH Candidates

To further investigate the structural diversity of RALPH candidates, a RALPH-related candidate set was first defined from the structural similarity network and subsequently refined. Briefly, candidates located in the core RALPH-enriched region of the network, together with additional candidates sharing homolog assignments to the same known powdery mildew effector set, were included, whereas candidates with pLDDT scores < 50 or lacking any RALPH-related features based on CATH, SCOPe, PDB, or known powdery mildew effector annotations were excluded.

The AlphaFold2-predicted structures of the selected RALPH candidates, together with known powdery mildew effectors assigned as RALPH-related in this study, were subjected to structure-based tree construction using FoldMason (release 4-dd3c235) with default settings [39]. The resulting topology was used to define major structural groups of RALPH candidates by recursive partitioning of deep internal splits in the tree topology. At each step, a split was accepted only when both descendant subtrees contained sufficient numbers of members, thereby avoiding over-fragmentation into many small terminal branches. This procedure defined major topology-defined structural groups, each representing a coherent major branch of the structure-based tree. The tree was visualized in iTOL 7 [40], and branch and ring colors were assigned according to structural group membership.

## 3. Results

### 3.1. Structural Overview of the Powdery Mildew Secretomes

**Figure 1:**
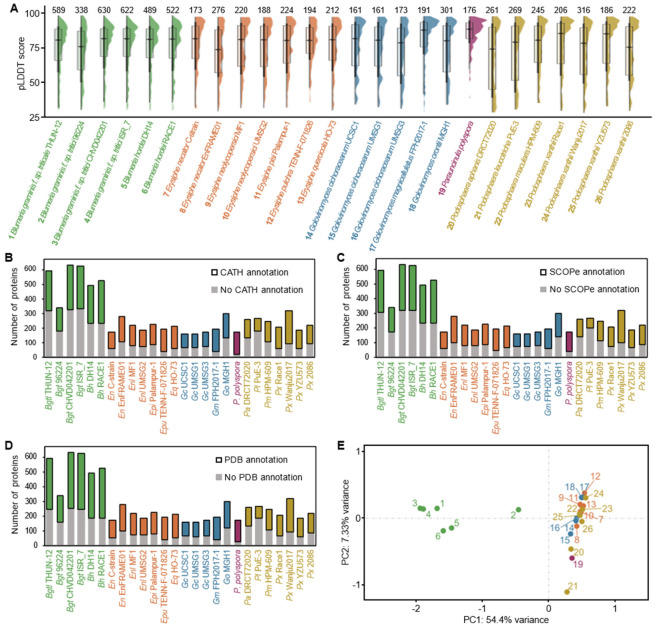
Overview of structure prediction confidence, annotation coverage, and structural relationships among powdery mildew secretomes. (**A**) Distribution of pLDDT scores for the top-ranked AlphaFold2 models of 7545 powdery mildew secretome candidates from 26 isolates. Numbers above the plots indicate the total number of secretome candidates in each isolate. Isolates are color-coded by genus: *Blumeria* (green), *Erysiphe* (orange), *Golovinomyces* (blue), *Parauncinula* (magenta), and *Podosphaera* (yellow). (**B**–**D**) Numbers of secretome candidates with and without retained structural annotations in the CATH (**B**), SCOPe (**C**), and PDB (**D**) databases, respectively. Outlined bars indicate annotated candidates, whereas gray bars indicate unannotated candidates. (**E**) Principal component analysis (PCA) of the 26 powdery mildew isolates based on the raw abundance matrix of 1807 Foldseek-derived structural clusters. Numbers 1–26 correspond to the isolate order used throughout the study. Percentages on the axes indicate the variance explained by each principal component.

To establish a structure-informed framework for comparative analysis, we predicted AlphaFold2 models for 7545 powdery mildew secretome candidates from 26 isolates representing five genera. Overall, the predicted models showed largely comparable pLDDT distributions across isolates (Figure 1A; Table S1). Most isolates had median pLDDT values around 80, indicating that the dataset was generally suitable for downstream structure-based analyses. Given that model confidence was generally acceptable and that some models with lower global scores could still contain well-predicted core structural regions, all 7545 predicted structures were retained for subsequent analyses rather than applying a fixed pLDDT cutoff.

We next examined how extensively these predicted structures could be related to existing structural resources. Across the 26 isolates, substantial fractions of secretome candidates received retained structural annotations in the CATH, SCOPe, and PDB databases, whereas the remaining candidates lacked retained matches under the applied criteria (Figure 1B–D; Table S1). Within individual isolates, the numbers of annotated candidates were generally similar between CATH and SCOPe, whereas PDB returned somewhat more matches. At the genus level, *Parauncinula* showed the highest annotation proportions across all three databases (CATH, 87.5%; SCOPe, 78.4%; PDB, 83.5%). Among the multi-isolate genera, *Erysiphe* had the highest overall annotation proportions (CATH, 65.8%; SCOPe, 65.6%; PDB, 67.6%). In contrast, *Blumeria* showed the lowest annotation proportions in CATH (49.1%) and SCOPe (50.4%), whereas *Podosphaera* showed the lowest proportion in PDB (57.7%). Together, these results indicate that powdery mildew secretomes contain both structurally conserved components that can be assigned to existing databases and a substantial fraction of candidates that remain uncharacterized by current structural references.

To further compare the global structural composition of the powdery mildew secretomes, all 7545 predicted structures were clustered into 1807 Foldseek-derived structural clusters, and their distributions were summarized across the 26 isolates (Table S4). Principal component analysis (PCA) based on the cluster abundance matrix separated *Blumeria* isolates from the other genera along principal component 1 (PC1), whereas the non-*Blumeria* genera occupied a more compact region with less separation along PC2 (Figure 1E). These results indicate broad structural commonality across genera while also suggesting pronounced separation of *Blumeria* from the other genera in PCA space based on raw cluster abundance. This pattern led us to investigate, in the analyses below, which structural components are widely conserved across powdery mildew secretomes and which contribute to lineage-associated differentiation.

### 3.2. Major CATH and SCOPe Fold/Domain Annotations Across Powdery Mildew Isolates

**Figure 2:**
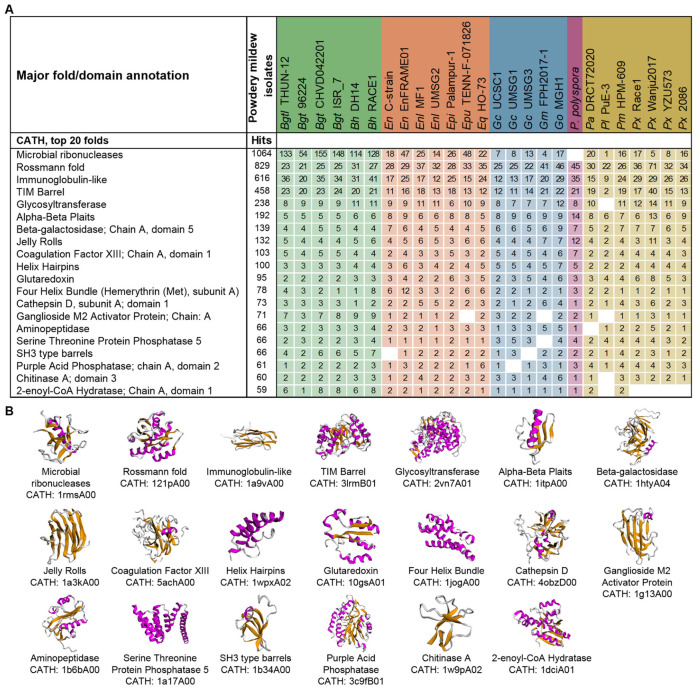
Major CATH fold/domain annotations detected in powdery mildew secretomes. (**A**) Distribution of the top 20 CATH fold/domain annotations among the 7545 powdery mildew secretome candidates across 26 isolates. Rows represent CATH annotations, columns represent isolates grouped by genus, and the hits column shows retained annotation-hit counts rather than unique-protein counts. (**B**) Representative structures of the CATH fold/domain annotations shown in panel A, as curated in the CATH database. Annotation names and representative CATH identifiers are shown below each structure.

**Figure 3:**
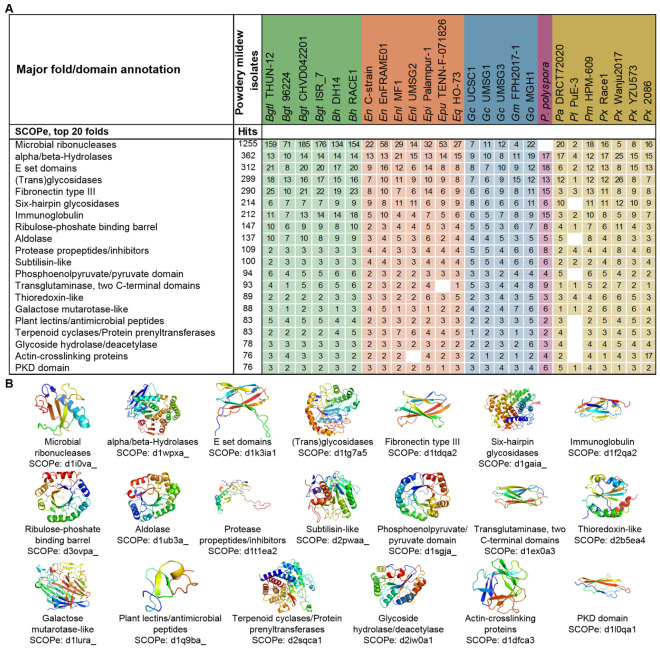
Major SCOPe fold/domain annotations detected in powdery mildew secretomes. (**A**) Distribution of the top 20 SCOPe fold/domain annotations among the 7545 powdery mildew secretome candidates across 26 isolates. Rows represent SCOPe annotations, columns represent isolates grouped by genus, and the hits column shows retained annotation-hit counts rather than unique-protein counts. (**B**) Representative structures of the SCOPe fold/domain annotations shown in panel A, as curated in the SCOPe database. Annotation names and representative SCOPe identifiers are shown below each structure.

To identify structurally conserved features shared across powdery mildew secretomes, we summarized the most abundant CATH and SCOPe fold/domain annotations across all isolates (Figure 2 and Figure 3; Tables S5 and S6). Because PDB entry annotations are heterogeneous, we did not generate a corresponding top-annotation summary for PDB; all retained PDB matches were recorded in Table S1 for reference.

In the CATH-based analysis, microbial ribonucleases represented the most abundant annotation, followed by Rossmann fold, immunoglobulin-like, and TIM barrel annotations (Figure 2A; Table S5). Other prominent CATH annotations included glycosyltransferase, alpha-beta plaits, jelly rolls, helix hairpins, glutaredoxin, aminopeptidase, SH3-type barrels, and other enzyme-associated folds. The SCOPe-based analysis yielded a similar pattern: microbial ribonucleases again ranked first, followed by alpha/beta-hydrolases, E set domains, (trans)glycosidases, fibronectin type III, and six-hairpin glycosidases (Figure 3A; Table S6). Additional prominent SCOPe annotations included immunoglobulin, ribulose-phosphate binding barrel, aldolase, subtilisin-like, thioredoxin-like, glycoside hydrolase/deacetylase, and PKD domain folds. Representative structures of these top annotations are shown in Figure 2B and Figure 3B.

Taken together, the CATH and SCOPe analyses revealed a consistent set of dominant fold/domain classes in powdery mildew secretomes. Both databases identified microbial ribonuclease annotations as the most prominent annotated feature, alongside abundant immunoglobulin/fibronectin-like, glycosidase-related, and enzyme-associated structural classes. Most top annotations were broadly conserved across genera, suggesting that powdery mildew secretomes share a common set of abundant structural features. In both databases, 19 of the top 20 annotations were present in all five genera; notably, microbial ribonucleases were the only top annotation absent in *Parauncinula polyspora* (Figure 2A and Figure 3A). At the same time, the relative abundance of this feature varied markedly among genera: microbial ribonuclease annotations accounted for 22.9% and 27.6% of secretome candidates in *Blumeria* in the CATH and SCOPe summaries, respectively, compared with 13.4% and 15.8% in *Erysiphe* and approximately 5% in both *Golovinomyces* and *Podosphaera*. These results support the presence of a broadly conserved structural core across powdery mildew secretomes, while also revealing lineage-associated differences in the relative abundance or absence of particular fold/domain categories.

### 3.3. Major Structural Clusters and Network Analysis Resolve the Core Architecture of Powdery Mildew Secretomes

**Figure 4:**
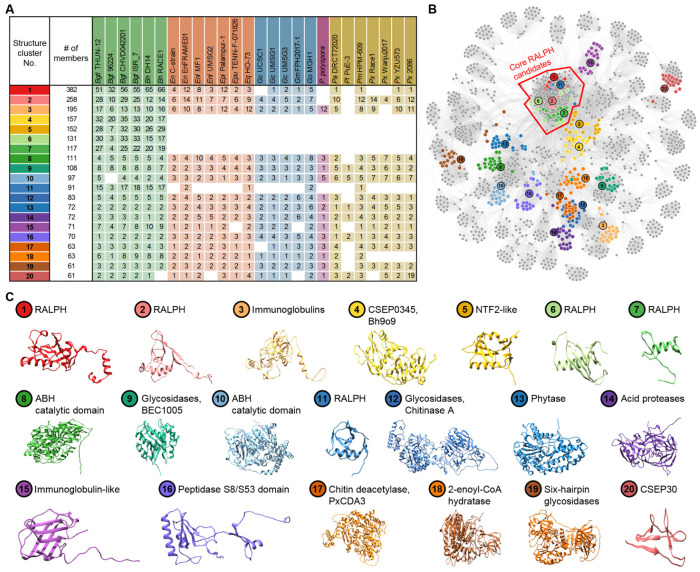
Major structural clusters and structural similarity network of powdery mildew secretomes. (**A**) Distribution of the top 20 Foldseek-derived structural clusters among the 7545 powdery mildew secretome candidates across 26 isolates. Rows represent structural clusters ranked by size, the # of members column shows the total number of proteins in each cluster, and columns represent isolates grouped by genus. (**B**) Structural similarity network was generated using representative proteins selected from the 92 largest structural clusters (20 highest-pLDDT proteins per cluster a total of 1840 representative proteins sample) and therefore represents major structural groups rather than the complete secretome dataset. Pairwise similarities were calculated using Foldseek alntmscore, and edges with alntmscore < 0.5 were removed. Nodes from the top 20 structural clusters are colored by cluster, whereas the remaining nodes are shown in gray. The red outline marks the region enriched in core RALPH candidates. (**C**) Representative structures of the top 20 structural clusters shown in panel A. These representatives were directly selected by Foldseek during structural clustering. Labels above the structures summarize annotation or known effector association information for the corresponding clusters.

To characterize the major structural components of powdery mildew secretomes, we summarized the 20 largest Foldseek-derived structural clusters across the 26 isolates (Figure 4A; Table S4). These clusters captured both broadly shared and lineage-enriched features. Among them, clusters 1, 2, 6, 7, and 11 were notable for their enrichment in microbial ribonuclease annotations and structural similarity to known RALPH effectors (AVR_A6_, AVR_A7_, AVR_A10_, AVR_A13_, AVR_A22_, AVRPM2, and BEC1054). These five clusters were therefore classified as RALPH-associated. *Parauncinula polyspora* lacked these RALPH-associated clusters. In addition, clusters 4, 5, 6, and 7 were detected only in *Blumeria*, indicating that this genus contains both *Blumeria*-specific RALPH-associated expansion (clusters 6 and 7) and additional non-RALPH structural diversification (clusters 4 and 5). In contrast, most clusters from 8 to 20 were broadly distributed across all five genera, with cluster 11 remaining part of the RALPH-associated component and absent from *Parauncinula polyspora*. This pattern supports the presence of a substantial shared structural component beyond the RALPH-enriched portion of the secretome.

To examine higher-order relationships among abundant structural clusters, we constructed a structural similarity network using representative proteins sampled from the 92 largest clusters (Figure 4B). Because the network was constructed using representative proteins from the largest structural clusters, it provides an overview of the major structural relationships within the secretome rather than a comprehensive depiction of all predicted proteins. The resulting network resolved multiple discrete modules rather than a continuous arrangement, indicating that major secretome structures partition into distinct structural neighborhoods. A conspicuous central region of the network was enriched in RALPH-associated clusters (1, 2, 6, 7, and 11), whereas non-RALPH clusters occupied more peripheral positions. The network also suggested outward structural diversification from this RALPH-enriched core, with adjacent clusters gradually extending into distinct neighborhoods. For instance, clusters 8 and 10 were both annotated as ABH catalytic domain-containing proteins yet formed distinct neighboring modules, illustrating structural divergence despite related annotation. Together, these patterns show that the core architecture of powdery mildew secretomes is organized around a prominent RALPH-centered component together with multiple additional structural communities.

Representative structures selected by Foldseek, together with the overall annotations of each cluster, are shown in Figure 4C. Beyond the RALPH-associated clusters, the major non-RALPH clusters included immunoglobulin/fibronectin-like, NTF2-like, glycosidase/chitinase-related, ABH catalytic domain-containing, phytase, acid protease, six-hairpin glycosidase, and CSEP30-like classes. Notably, many of these large non-RALPH clusters showed little or no structural similarity to currently identified known powdery mildew effectors (Table S1), indicating that substantial portions of the major cluster landscape remain structurally defined but functionally uncharacterized.

Together, these results show that the largest structural clusters, as represented in this sampled network analysis, define a central portion of the core architecture of powdery mildew secretomes. This architecture comprises a central RALPH-centered component, several *Blumeria*-specific structural groups, and a broader set of conserved non-RALPH clusters shared across genera. These patterns motivated a focused analysis of the RALPH candidate repertoire in the following section.

### 3.4. Structure-Based Grouping Resolves the Diversity of RALPH Candidates

**Figure 5:**
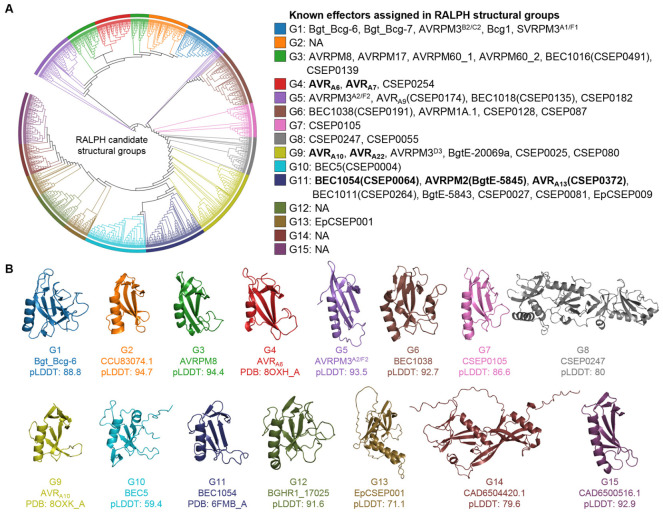
Structure-based grouping of RALPH candidates and assignment of known powdery mildew effectors. (**A**) Structure-based tree of RALPH candidate proteins subdivided into 15 structural groups (G1–G15). Branches and outer rings are colored by group. Known powdery mildew effectors assigned to each group are listed on the right; effector names in bold indicate those with experimentally determined PDB structures. NA indicates that no previously characterized powdery mildew effector was assigned to that group. (**B**) Representative structures of the 15 RALPH structural groups shown in panel (**A**). PDB structures were used when available; otherwise, the highest-pLDDT AlphaFold2 model from a previously characterized powdery mildew effector in the group was selected, or, if none was present, the highest-pLDDT AlphaFold2 model in the group. Colors correspond to the group colors used in panel (**A**).

Because RALPH-related proteins formed a central component of the powdery mildew secretome structural landscape, we analyzed this subset in detail (Figure 5). RALPH candidates were first collected from the core RALPH-enriched region of the structural similarity network, corresponding to 21 clusters in Figure 4B (clusters 1, 2, 6, 7, 11, 25, 26, 30, 32, 34, 39, 43, 45, 50, 51, 54, 62, 64, 76, 84, and 87). This core region was supported by structural similarity to well-characterized RALPH effector structures, including AVR_A6_, AVR_A7_, AVR_A10_, AVR_A13_, AVR_A22_, AVRPM2, and BEC1054, as well as to additional known powdery mildew effectors placed in the same RALPH structural space in this study (Table S2). Because the network analysis included only the 92 largest clusters, we also incorporated clusters 114, 121, 146, and 283 when they contained candidates with similarity to the same RALPH-related effector set. After excluding candidates with pLDDT scores below 50 and those lacking any RALPH-related annotation features, 1502 predicted proteins were retained. These proteins, together with experimentally determined PDB chains from known RALPH effectors, were subjected to FoldMason to generate a structure-based tree for grouping analysis.

The resulting structure-based tree resolved multiple major branches within the RALPH repertoire, and subsequent topology-based partitioning defined 15 structural groups (G1–G15) (Figure 5A). Known and experimentally characterized powdery mildew effectors were assigned to several of these groups, linking the broader RALPH candidate space to previously characterized effector proteins. For instance, AVR_A6_ and AVR_A7_ clustered together, AVR_A10_ and AVR_A22_ formed another branch, and BEC1054, AVRPM2, and AVR_A13_ grouped into a distinct branch. Other groups contained combinations of previously characterized effectors such as BEC1038 (CSEP0191), AVRPM1A.1, CSEP0105, CSEP0247, CSEP0055, and EpCSEP001. In contrast, several groups lacked any currently assigned effectors, indicating that substantial portions of the RALPH structural space remain functionally uncharacterized.

Representative structures of the 15 groups highlighted both the shared architectural framework and the substantial structural diversification within the broader RALPH repertoire (Figure 5B). Some groups were represented by experimentally determined structures, whereas others were represented by high-confidence AlphaFold2 models. Together, these results show that predicted RALPH candidates do not form a single uniform structural class, but instead constitute a diverse and recurrent repertoire that can be partitioned into multiple topology-defined groups.

### 3.5. Structural Features Not Detected in the Sampled Blumeria Datasets Reveal Lineage-Associated Differences Among Powdery Mildew Secretomes

**Figure 6:**
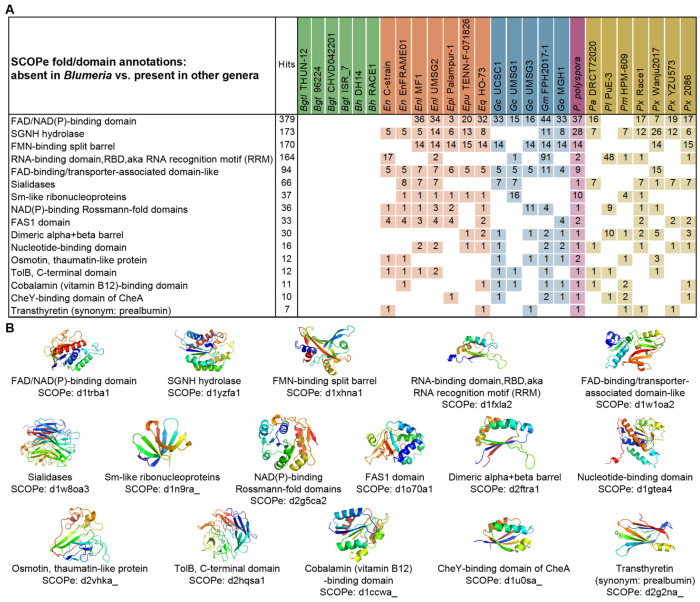
SCOPe fold/domain annotations not detected in the sampled *Blumeria* datasets but present in other powdery mildew genera. (**A**) Distribution of SCOPe fold/domain annotations not detected in the sampled *Blumeria* datasets but present in other powdery mildew genera. Rows represent SCOPe annotations, columns represent isolates grouped by genus, and the hits column shows the total number of retained annotation hits for each annotation. (**B**) Representative structures of the SCOPe fold/domain annotations shown in panel A, as curated in the SCOPe database. Annotation names and representative SCOPe identifiers are shown below each structure.

The preceding analyses showed that *Blumeria* secretomes are clearly separated from those of the other genera in the structural cluster-based PCA (Figure 1E) and include several expanded or *Blumeria*-specific structural components, including RALPH-associated clusters and additional lineage-enriched clusters such as clusters 4 and 5 (Figure 4). However, this observation should be interpreted cautiously because *Blumeria* represents monocot-associated powdery mildews in the present dataset, whereas the remaining genera are predominantly dicot-associated. Consequently, some of the observed structural differences may reflect host-associated evolutionary patterns in addition to genus-level divergence. We therefore asked whether structural features present in the other powdery mildew genera were not detected in the sampled *Blumeria* datasets. To address this, we examined fold/domain categories detected in *Erysiphe*, *Golovinomyces*, *Parauncinula*, and *Podosphaera* but were not detected in the sampled *Blumeria* datasets (Figure 6). This analysis identified a distinct set of structural classes not detected in the sampled *Blumeria* datasets.

Among the most prominent annotations not detected in the sampled *Blumeria* datasets were FAD/NAD(P)-binding domain, SGNH hydrolase, FMN-binding split barrel, RNA-binding domain/RRM, and FAD-binding/transporter-associated domain-like folds (Figure 6A). Additional categories included sialidases, Sm-like ribonucleoproteins, NAD(P)-binding Rossmann-fold domains, FAS1 domain, dimeric alpha+beta barrel, nucleotide-binding domain, osmotin/thaumatin-like protein, TolB C-terminal domain, cobalamin-binding domain, CheY-binding domain of CheA, and transthyretin-like folds. Representative structures of these categories are shown in Figure 6B. Notably, all of these categories were detected in the dicot-associated powdery mildew genera included in this study, but not in *Blumeria*.

Together, these results indicate that lineage-associated divergence in powdery mildew secretomes involves not only expansion of particular structural groups in *Blumeria*, but also the absence of multiple fold/domain categories retained in the dicot-associated genera. Given that the *Blumeria* isolates analyzed here are monocot-associated, whereas the remaining genera in this dataset are dicot-associated, this pattern may also be consistent with structural differences associated with host lineage.

### 3.6. Structural Comparison with Defined Fungal Effector Structural Families Identifies MoHrip2-like as the Major Shared Fold

**Figure 7:**
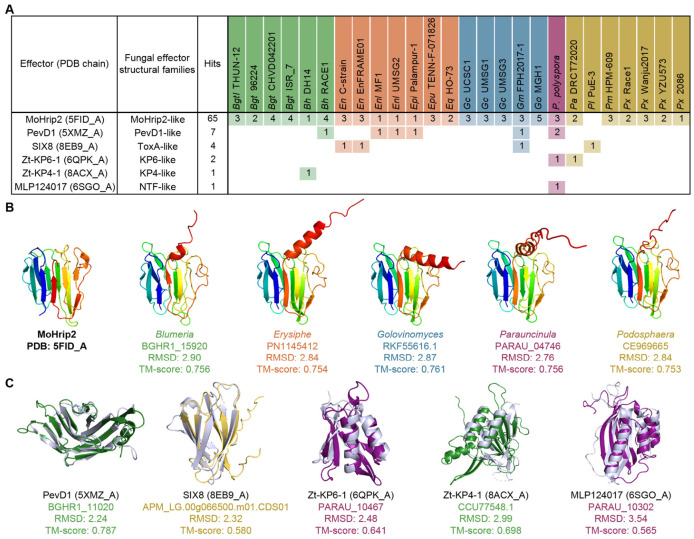
Structural similarity of powdery mildew secretome candidates to defined fungal effector structural families. (**A**) Distribution of powdery mildew secretome candidates assigned to defined fungal effector structural families based on comparisons with reference effector PDB chains. Rows list the reference effector PDB chains and their corresponding fungal effector structural families, columns represent isolates grouped by genus, and the hits column shows the total number of assigned candidates for each reference effector PDB chain. (**B**) Representative MoHrip2-like secretome candidates from the five powdery mildew genera. The MoHrip2 reference structure (PDB: 5FID_A) is shown on the left, followed by one representative candidate from each genus. RMSD and TM-score values were obtained by US-align comparisons between each candidate and the MoHrip2 reference structure. (**C**) Representative structural superpositions of powdery mildew secretome candidates and reference fungal effector structures for the PevD1-like, ToxA-like, KP6-like, KP4-like, and NTF-like families. Powdery mildew candidates are colored by genus, and the reference structures are shown in gray. RMSD and TM-score values were obtained by US-align comparisons between each candidate and the corresponding reference structure.

We next compared powdery mildew secretome structures against a curated reference set of defined fungal effector structural families (Figure 7; Table S3). Of the 13 reference families examined, retained matches were detected only for MoHrip2-like, PevD1-like, ToxA-like, KP6-like, KP4-like, and NTF-like families, whereas no retained matches were found for AvrSr35-like, AvrSr50-like, FOLD, LARS, MAX, Tin2-like, or ZiF. Thus, powdery mildew secretomes share structural similarity with only a limited subset of currently defined fungal effector structural families.

Among the matched families, MoHrip2-like structures were the most prominent, with 65 assigned candidates distributed across all five powdery mildew genera (Figure 7A). The remaining matched families, including PevD1-like, ToxA-like, KP6-like, KP4-like, and NTF-like, were each represented by only a small number of candidates. Representative comparisons showed that one MoHrip2-like candidate was identified from each powdery mildew genus, and all showed clear structural similarity to the MoHrip2 reference (Figure 7B). Representative superpositions further supported structural similarity between selected powdery mildew candidates and reference structures from the other matched fungal effector structural families (Figure 7C). Together, these results identify MoHrip2-like as the most prominent shared fold among the defined fungal effector structural families matched in powdery mildew secretomes, while indicating that only a small number of additional defined fungal effector families are represented by secretome candidates in this dataset.

## 4. Discussion

This study extends our previous sequence-based comparative analysis of powdery mildew secretomes into the structural dimension [28]. By integrating AlphaFold2-based structure prediction with structural annotation, clustering, and network analysis, we compared secretome architecture across 26 isolates representing five powdery mildew genera, including several genera whose secretomes remain much less characterized than those of *Blumeria*. Two main patterns emerged. First, powdery mildew secretomes retain a broadly shared structural foundation across genera. Second, this conserved background is accompanied by marked lineage-associated differentiation, with *Blumeria* showing both expansion of RALPH-associated components and non-detection of multiple fold/domain categories retained in the dicot-associated genera. Together, these findings place powdery mildew secretome diversity in a comparative structural framework and provide a basis for interpreting both conserved and lineage-specific features of effector repertoires.

One of the clearest outcomes is the presence of a broadly shared structural core across powdery mildew secretomes. Across the five genera examined, the dominant CATH and SCOPe annotations were largely conserved, with microbial ribonucleases, immunoglobulin/fibronectin-like, glycosidase-related, and other enzyme-associated structural classes repeatedly represented among the top fold/domain categories (Figure 2 and Figure 3). This pattern indicates that powdery mildew secretomes retain a common structural foundation despite substantial taxonomic divergence. The same conclusion is supported by the major structural clusters, many of which were broadly distributed across genera (Figure 4). Importantly, this shared structural foundation is defined at the level of the broader secretome candidate set and should not be interpreted as implying that all recurrent structural classes represent conserved effector families. In a broader fungal context, the prominence of MoHrip2-like matches across all five genera further suggests that some effector folds recur across distinct phytopathogenic lineages (Figure 7). This interpretation is consistent with the structurally characterized MoHrip2 protein from *Magnaporthe oryzae* [41], with recent structure-guided studies showing that certain effector folds recur across multiple fungal taxa even when sequence similarity is limited [29,37], and with the identification of MoHrip2-like effector candidates across multiple *Puccinia striiformis* races [42].

Compared with our previous sequence-based comparative analysis [28], the present structure-based framework provides a complementary view of secretome diversification. In both studies, *Blumeria* was clearly separated from the remaining genera, indicating that its distinctiveness is robust at both the sequence and structural levels. An additional consideration is that *Blumeria* differs from the other sampled genera not only taxonomically but also in host association, as it infects monocots while the remaining genera are largely associated with dicots. Therefore, part of the observed structural differentiation may reflect host-driven evolutionary adaptation rather than genus-level divergence alone. Additional sampling of monocot-associated powdery mildew species will be required to disentangle these factors. The structural cluster-based PCA placed the non-*Blumeria* genera in a relatively more compact region of ordination space (Figure 1E), suggesting that the pattern of structural differentiation is not identical to that inferred from sequence-based divergence. Because this PCA was based on raw cluster abundance, and secretome sizes differ substantially among isolates ranging from 161 to 630 proteins, the observed separation of *Blumeria* from other genera along PC1 reflects a combination of genuine structural repertoire differences and overall secretome expansion in this genus. While raw abundance PCA effectively captures the distinctiveness of *Blumeria* at the level of absolute cluster counts, it should be interpreted primarily as a summary of overall repertoire composition rather than as a size-normalized comparison. The structural framework developed here therefore does not replace sequence-based analysis, but adds another level for distinguishing deeper structural conservation from clearer lineage-associated differentiation. Future studies employing size-normalized approaches may provide additional insights into compositional differences independent of secretome size.

The prominence of RALPH-related structures observed in *Blumeria* is consistent with numerous previous studies describing the expansion and diversification of RALPH effectors in cereal powdery mildews. Therefore, this finding primarily serves as an internal validation of the structure-based analytical framework employed here rather than a novel biological observation. RALPH-related proteins emerged as a central structural component of powdery mildew secretomes. This broader RALPH-centered interpretation is also consistent with earlier work in cereal mildew and with subsequent identification of RALPH-related candidates in *Erysiphe pisi*, including effectors such as EpCSEP001 and EpCSEP009 that are represented in our structure-based grouping framework [10,23]. This was evident not only from the prominence of microbial ribonuclease annotations in the CATH and SCOPe summaries (Figure 2 and Figure 3), but also from the concentration of RALPH-associated clusters within the largest structural groups and the core of the structural similarity network (Figure 4). At the same time, the RALPH repertoire was not structurally uniform. Structure-based grouping resolved this component into multiple topology-defined groups, several of which could be linked to previously characterized powdery mildew effectors, whereas others remained structurally coherent but lacked functional assignment (Figure 5). These results extend the current view of RALPH effectors from a limited set of individually characterized proteins to a broader structural repertoire that includes both conserved and structurally diverse components across powdery mildew secretomes. This interpretation is also consistent with earlier evidence for large-scale expansion and diversification of effector candidates in *Blumeria* [10]. The structural distinctiveness of *Blumeria*, while potentially influenced by its unique representation as the monocot-associated lineage in the current dataset, appears to be shaped not only by expansion of particular structural groups, but also by absence of others. *Blumeria* contained lineage-enriched components within the major cluster landscape, including *Blumeria*-specific RALPH-associated expansion and additional non-RALPH clusters such as clusters 4 and 5 (Figure 4), consistent with earlier evidence for large-scale diversification of effector candidates in this genus [10]. Population-genomic analyses further indicate that wheat powdery mildew lineages are evolutionarily dynamic at global scale, reinforcing the view that *Blumeria* diversification should be interpreted in a broader genomic context [43]. At the same time, multiple fold/domain categories detected in the other genera were not detected in the sampled *Blumeria* datasets (Figure 6). This combined pattern suggests that *Blumeria* divergence is better understood as broader remodeling of secretome structural composition rather than as amplification of a single effector type. These lineage-associated differences also raise questions about the role of host association in shaping effector repertoires. The *Blumeria* materials included here were limited to triticale-, wheat-, and barley-infecting lineages, whereas the other genera infect a diverse range of dicot hosts. Accordingly, the observed combination of *Blumeria*-enriched RALPH-associated expansion and fold/domain categories not detected in the sampled *Blumeria* datasets may be consistent with host-lineage-associated structural differentiation, but broader sampling of monocot-associated powdery mildews will be needed to evaluate this possibility more fully.

Beyond confirming previously reported RALPH expansions, the principal contribution of this study lies in the comprehensive structural comparison of secretomes across five powdery mildew genera. This analysis revealed both conserved and lineage-associated structural clusters, including several non-RALPH groups that would be difficult to identify through sequence-based approaches alone. These findings expand our understanding of powdery mildew secretome diversity and demonstrate the value of structure-guided analyses for uncovering evolutionary relationships among highly divergent candidate effectors.

Several limitations should be acknowledged. Importantly, the vast majority of proteins analyzed in this study represent computationally predicted candidate secretomes. Although structural similarity can provide valuable clues regarding evolutionary relationships and potential functions, these predictions do not constitute experimental evidence of effector activity. First, the predicted structures, while generally supported by high pLDDT scores and by agreement with selected experimentally determined reference structures, remain computational models awaiting experimental confirmation. Second, the structural annotation databases used (CATH, SCOPe, and PDB) are biased toward known structures and may therefore underestimate novel folds in powdery mildew secretomes. Third, our analyses focused on structural similarity rather than functional activity, and structural similarity does not necessarily imply functional equivalence. Finally, taxon sampling was uneven across genera. *Parauncinula polyspora* was represented by a single available dataset, and the *Blumeria* materials were restricted to a narrow subset of monocot-associated lineages because broader sampling of monocot-associated powdery mildews was constrained by the scarcity of available genomic resources. Therefore, conclusions regarding *Blumeria*-specific structural features should be considered provisional until additional monocot-associated powdery mildew genomes become available. This is particularly relevant because *P. polyspora* lacked microbial ribonuclease annotations in the major CATH and SCOPe summaries and was absent from the major RALPH-associated clusters, although this observation should be interpreted cautiously given the limited sampling. Nevertheless, the structural groups and candidate effectors identified here provide a prioritized resource for downstream functional characterization. Experimental validation of representative candidates from large structural clusters lacking matches to known powdery mildew effectors could yield new insights into powdery mildew virulence mechanisms. Broader comparative analyses incorporating more diverse powdery mildew species and other fungal pathogens will also help determine whether the patterns observed here reflect general principles of effector evolution or more lineage-specific features.

In summary, this study shows that comparative structural analysis can clarify both conserved and lineage-associated features of powdery mildew secretomes that are not fully captured by sequence-based analysis alone. In particular, the results highlight the central role of RALPH-related proteins in secretome organization, the marked structural distinctiveness of *Blumeria*, and the broader relevance of selected shared fungal effector-like folds detected in the reference-family comparison, particularly MoHrip2-like. Together, these findings refine the structural framework of powdery mildew effector biology and provide a basis for future studies on conserved and lineage-specific mechanisms of pathogenicity.

## 5. Conclusions

This study establishes a structure-based comparative framework for powdery mildew secretomes across 26 isolates from five genera. Our analyses reveal a broadly conserved structural core shared across genera, alongside pronounced lineage-associated divergence, with *Blumeria* showing both expansion of RALPH-associated components and non-detection of multiple fold/domain categories that were retained in the sampled dicot-associated genera, a pattern that may also be consistent with host lineage-associated differences in secretome composition. RALPH-related proteins form a structurally diverse repertoire that partitions into multiple topology-defined groups, while comparison with defined fungal effector families identifies MoHrip2-like as the most prominent shared fold. While our analyses confirm the prominent role of RALPH-related proteins in *Blumeria* secretomes, the broader novelty of this study lies in revealing conserved and lineage-specific structural patterns across multiple powdery mildew genera at an unprecedented scale. Together, these findings provide a systematic structural view of powdery mildew effector biology and establish a prioritized resource for future functional studies of conserved and lineage-specific candidate effectors, whose predicted activities and biological roles remain to be experimentally validated.

## Data Availability

The AlphaFold2-predicted structures of 7545 powdery mildew secretome candidates and 75 known powdery mildew effectors are openly available in Zenodo at https://zenodo.org/records/19402368 (accessed on 3 March 2026). The original contributions presented in this study are included in the article/Supplementary Material. Further inquiries can be directed to the corresponding authors.

## Supplementary Materials

The following supporting information can be downloaded at: https://www.mdpi.com/article/10.3390/pathogens15060612/s1, Table S1: Structural features and annotation summary of 7545 powdery mildew secretome candidates; Table S2: Known powdery mildew effectors used as reference structures in this study; Table S3: Defined fungal effector structural families used for structural similarity searches; Table S4: Distribution of Foldseek-derived structural clusters across 26 powdery mildew isolates; Table S5: CATH fold/domain annotation distribution across 26 powdery mildew isolates; Table S6: SCOPe fold/domain annotation distribution across 26 powdery mildew isolates.

## References

[1] Bourras S., Praz C.R., Spanu P.D., Keller B. (2018). Cereal powdery mildew effectors: A complex toolbox for an obligate pathogen. Curr. Opin. Microbiol..

[2] Kusch S., Qian J., Loos A., Kümmel F., Spanu P.D., Panstruga R. (2024). Long-term and rapid evolution in powdery mildew fungi. Mol. Ecol..

[3] Hückelhoven R., Panstruga R. (2011). Cell biology of the plant-powdery mildew interaction. Curr. Opin. Plant Biol..

[4] Mapuranga J., Zhang L., Zhang N., Yang W. (2022). The haustorium: The root of biotrophic fungal pathogens. Front. Plant Sci..

[5] Li G., Newman M., Yu H., Rashidzade M., Martínez-Soto D., Caicedo A., Allen K.S., Ma L.J. (2024). Fungal effectors: Past, present, and future. Curr. Opin. Microbiol..

[6] Spanu P.D., Abbott J.C., Amselem J., Burgis T.A., Soanes D.M., Stüber K., Ver Loren van Themaat E., Brown J.K.M., Butcher S.A., Gurr S.J. (2010). Genome expansion and gene loss in powdery mildew fungi reveal tradeoffs in extreme parasitism. Science.

[7] Wicker T., Oberhaensli S., Parlange F., Buchmann J.P., Shatalina M., Roffler S., Ben-David R., Doležel J., Šimková H., Schulze-Lefert P. (2013). The wheat powdery mildew genome shows the unique evolution of an obligate biotroph. Nat. Genet..

[8] Bilstein-Schloemer M., Müller M.C., Saur I.M.L. (2025). Technical advances drive the molecular understanding of effectors from wheat and barley powdery mildew fungi. Mol. Plant Microbe Interact..

[9] Spanu P.D. (2017). Cereal immunity against powdery mildews targets RNase-like proteins associated with haustoria (RALPH) effectors evolved from a common ancestral gene. New Phytol..

[10] Pedersen C., Ver Loren van Themaat E., McGuffin L.J., Abbott J.C., Burgis T.A., Barton G., Bindschedler L.V., Lu X., Maekawa T., Weßling R. (2012). Structure and evolution of barley powdery mildew effector candidates. BMC Genom..

[11] Manser B., Koller T., Praz C.R., Roulin A.C., Zbinden H., Arora S., Steuernagel B., Wulff B.B.H., Keller B., Sánchez-Martín J. (2021). Identification of specificity-defining amino acids of the wheat immune receptor Pm2 and powdery mildew effector AvrPm2. Plant J..

[12] Pennington H.G., Jones R., Kwon S., Bonciani G., Thieron H., Chandler T., Luong P., Morgan S.N., Przydacz M., Bozkurt T. (2019). The fungal ribonuclease-like effector protein CSEP0064/BEC1054 represses plant immunity and interferes with degradation of host ribosomal RNA. PLoS Pathog..

[13] Cao Y., Kümmel F., Logemann E., Gebauer J.M., Lawson A.W., Yu D., Uthoff M., Keller B., Jirschitzka J., Baumann U. (2023). Structural polymorphisms within a common powdery mildew effector scaffold as a driver of coevolution with cereal immune receptors. Proc. Natl. Acad. Sci. USA.

[14] Lawson A.W., Flores-Ibarra A., Cao Y., An C., Neumann U., Gunkel M., Saur I.M.L., Chai J., Behrmann E., Schulze-Lefert P. (2025). The barley MLA13-AVR_A13_ heterodimer reveals principles for immunoreceptor recognition of RNase-like powdery mildew effectors. EMBO J..

[15] Saur I.M., Bauer S., Kracher B., Lu X., Franzeskakis L., Müller M.C., Sabelleck B., Kümmel F., Panstruga R., Maekawa T. (2019). Multiple pairs of allelic MLA immune receptor-powdery mildew AVR_A_ effectors argue for a direct recognition mechanism. eLife.

[16] Pennington H.G., Gheorghe D.M., Damerum A., Pliego C., Spanu P.D., Cramer R., Bindschedler L.V. (2016). Interactions between the powdery mildew effector BEC1054 and barley proteins identify candidate host targets. J. Proteome Res..

[17] Müller M.C., Praz C.R., Sotiropoulos A.G., Menardo F., Kunz L., Schudel S., Oberhänsli S., Poretti M., Wehrli A., Bourras S. (2019). A chromosome-scale genome assembly reveals a highly dynamic effector repertoire of wheat powdery mildew. New Phytol..

[18] Seong K., Krasileva K.V. (2021). Computational structural genomics unravels common folds and novel families in the secretome of fungal phytopathogen *Magnaporthe oryzae*. Mol. Plant Microbe Interact..

[19] Jones D.A.B., Raffaele S. (2025). Phytopathogen effector biology in the burgeoning AI era. Annu. Rev. Phytopathol..

[20] Lahfa M., Barthe P., de Guillen K., Cesari S., Raji M., Kroj T., Le Naour-Vernet M., Hoh F., Gladieux P., Roumestand C. (2024). The structural landscape and diversity of *Pyricularia oryzae* MAX effectors revisited. PLoS Pathog..

[21] De la Concepcion J.C., Langner T., Fujisaki K., Yan X., Were V., Lam A.H.C., Saado I., Brabham H.J., Win J., Yoshida K. (2024). Zinc-finger (ZiF) fold secreted effectors form a functionally diverse family across lineages of the blast fungus *Magnaporthe oryzae*. PLoS Pathog..

[22] Yu D.S., Outram M.A., Smith A., McCombe C.L., Khambalkar P.B., Rima S.A., Sun X., Ma L., Ericsson D.J., Jones D.A. (2024). The structural repertoire of *Fusarium oxysporum* f. sp. *lycopersici* effectors revealed by experimental and computational studies. eLife.

[23] Sharma G., Aminedi R., Saxena D., Gupta A., Banerjee P., Jain D., Chandran D. (2019). Effector mining from the *Erysiphe pisi* haustorial transcriptome identifies novel candidates involved in pea powdery mildew pathogenesis. Mol. Plant Pathol..

[24] Polonio Á., Fernández-Ortuño D., de Vicente A., Pérez-García A. (2021). A haustorial-expressed lytic polysaccharide monooxygenase from the cucurbit powdery mildew pathogen *Podosphaera xanthii* contributes to the suppression of chitin-triggered immunity. Mol. Plant Pathol..

[25] Martínez-Cruz J.M., Polonio Á., Ruiz-Jiménez L., Vielba-Fernández A., Hierrezuelo J., Romero D., de Vicente A., Fernández-Ortuño D., Pérez-García A. (2022). Suppression of chitin-triggered immunity by a new fungal chitin-binding effector resulting from alternative splicing of a chitin deacetylase gene. J. Fungi.

[26] Mu B., Chen J., Wang H., Kong W., Fan X., Wen Y.Q. (2022). An effector CSEP087 from *Erysiphe necator* targets arginine decarboxylase VviADC to regulate host immunity in grapevine. Sci. Hortic..

[27] Mu B., Teng Z., Tang R., Lu M., Chen J., Xu X., Wen Y.Q. (2023). An effector of *Erysiphe necator* translocates to chloroplasts and plasma membrane to suppress host immunity in grapevine. Hortic. Res..

[28] Ali N., Wu N., Akkaya M.S. (2026). Sequence-based comparative secretome analysis reveals conserved core effectors and host lineage-specific divergence between monocot- and dicot-associated powdery mildew lineages. Front. Plant Sci..

[29] Seong K., Krasileva K.V. (2023). Prediction of effector protein structures from fungal phytopathogens enables evolutionary analyses. Nat. Microbiol..

[30] Jumper J., Evans R., Pritzel A., Green T., Figurnov M., Ronneberger O., Tunyasuvunakool K., Bates R., Žídek A., Potapenko A. (2021). Highly accurate protein structure prediction with AlphaFold. Nature.

[31] Mirdita M., Schütze K., Moriwaki Y., Heo L., Ovchinnikov S., Steinegger M. (2022). ColabFold: Making protein folding accessible to all. Nat. Methods.

[32] Sillitoe I., Bordin N., Dawson N., Waman V.P., Ashford P., Scholes H.M., Pang C.S.M., Woodridge L., Rauer C., Sen N. (2021). CATH: Increased structural coverage of functional space. Nucleic Acids Res..

[33] Chandonia J.M., Guan L., Lin S., Yu C., Fox N.K., Brenner S.E. (2022). SCOPe: Improvements to the structural classification of proteins—Extended database to facilitate variant interpretation and machine learning. Nucleic Acids Res..

[34] Berman H.M., Westbrook J., Feng Z., Gilliland G., Bhat T.N., Weissig H., Shindyalov I.N., Bourne P.E. (2000). The Protein Data Bank. Nucleic Acids Res..

[35] van Kempen M., Kim S.S., Tumescheit C., Mirdita M., Lee J., Gilchrist C.L.M., Söding J., Steinegger M. (2024). Fast and accurate protein structure search with Foldseek. Nat. Biotechnol..

[36] Bastian M., Heymann S., Jacomy M. (2009). Gephi: An open source software for exploring and manipulating networks. Proc. Int. AAAI Conf. Web Soc. Media.

[37] Le Naour-Vernet M., Lahfa M., Maidment J.H.R., Padilla A., Roumestand C., de Guillen K., Kroj T., Césari S. (2025). Structure-guided insights into the biology of fungal effectors. New Phytol..

[38] Zhang C., Shine M., Pyle A.M., Zhang Y. (2022). US-align: Universal structure alignments of proteins, nucleic acids, and macromolecular complexes. Nat. Methods.

[39] Gilchrist C.L.M., Mirdita M., Steinegger M. (2026). Multiple protein structure alignment at scale with FoldMason. Science.

[40] Letunic I., Bork P. (2024). Interactive Tree of Life (iTOL) v6: Recent updates to the phylogenetic tree display and annotation tool. Nucleic Acids Res..

[41] Liu M., Duan L., Wang M., Zeng H., Liu X., Qiu D. (2016). Crystal structure analysis and the identification of distinctive functional regions of the protein elicitor Mohrip2. Front. Plant Sci..

[42] Asghar R., Wu N., Ali N., Wang Y., Akkaya M. (2025). Computational studies reveal structural characterization and novel families of *Puccinia striiformis* f. sp. *tritici* effectors. PLoS Comput. Biol..

[43] Sotiropoulos A.G., Arango-Isaza E., Ban T., Barbieri C., Bourras S., Cowger C., Czembor P.C., Ben-David R., Dinoor A., Ellwood S.R. (2022). Global genomic analyses of wheat powdery mildew reveal association of pathogen spread with historical human migration and trade. Nat. Commun..

